# Human-AI Interaction in Low- and Middle-Income Countries: Qualitative Study of How Local Human Factors Influence AI Development and Deployment

**DOI:** 10.2196/78649

**Published:** 2026-06-03

**Authors:** Evangelia Baka, Niklas Krischer, Udani De Silva, Yi-Roe Tan, Peiling Yap, Brian Li Han Wong

**Affiliations:** 1 Virtual Medicine Centre, University Hospital of Geneva, Geneva, Switzerland; 2 Faculty of Health, Medicine and Life Sciences, Maastricht University, Maastricht, The Netherlands; 3 Centre for Population Health Sciences, Usher Institute, University of Edinburgh, Edinburgh, United Kingdom; 4 HealthAI - The Global Agency for Responsible AI in Health, Geneva, Switzerland; 5 Department of International Health, Care and Public Health Research Institute, Maastricht University, Universiteitssingel 40, Maastricht, 6229 ER, The Netherlands; 6 Digital Health and Artificial Intelligence Section, European Public Health Association, Utrecht, The Netherlands

**Keywords:** human-AI collaboration, AI governance, AI ethics, participatory design, cultural adaptation, responsible AI, LMICs, low- and middle-income countries

## Abstract

**Background:**

Artificial intelligence (AI) is rapidly transforming health care and health research, offering new opportunities for improving efficiency, accessibility, and equity. However, the ethical, societal, and regulatory challenges of AI development and deployment are particularly pronounced in low- and middle-income countries (LMICs). While existing literature often emphasizes high-level ethical principles or technical frameworks, there is a notable gap in empirical, qualitative research that centers on human involvement and sociocultural dynamics throughout the AI lifecycle in LMIC contexts.

**Objective:**

This study addresses this gap by exploring the role of human involvement across the AI lifecycle, examining how cultural, societal, and governance factors influence AI perceptions and expectations in LMICs.

**Methods:**

We conducted 21 qualitative online interviews with AI researchers and innovators across MENA (Middle East and North Africa), Africa, Latin America, and Asia. A semistructured interview guide informed by the KAP (knowledge, attitude, and practice) framework was used. A thematic analysis approach was used for coding and analyzing the transcripts.

**Results:**

We identified five key themes: (1) the necessity of human oversight and the readiness required to support it, (2) the need for AI ethics training, (3) the importance of developing AI systems tailored to local realities, (4) the role of human-centered AI governance, and (5) the value of securing multidisciplinary teams. Findings highlight critical gaps in AI literacy, ethical governance, and interdisciplinary collaboration, emphasizing that AI solutions must be co-designed with local communities to be culturally and contextually relevant.

**Conclusions:**

This study underscores the urgent need for participatory AI development in LMICs and calls for investment in AI education, ethical oversight, and inclusive governance frameworks to ensure that AI serves as a tool for social equity rather than exclusion.

## Introduction

The artificial intelligence (AI) revolution has permeated and transformed numerous industries across the world, including health care and health research. In this paper, we focus specifically on AI applications in health care, hereafter referred to as “health care AI,” which encompass digital systems and algorithms designed to support diagnosis, treatment decision-making, and health services delivery. Through AI, health care has the potential to become more accessible, cost-efficient, and adaptable, with its implementation in various applications ranging from personalized health advice to public health education. The goal of AI technologies in this context is not to replace human judgment but to augment the decision-making process by analyzing data and generating insights that support improved clinical and organizational outcomes [1]. Therefore, human decision-making across the entire AI lifecycle, from data collection and model development to deployment and evaluation, is crucial to ensure that AI serves health care goals ethically and effectively. Each of the AI lifecycle stages requires human oversight and contextual understanding to mitigate risks such as bias, inaccuracy, or inequity.

In particular, low- and middle-income countries (LMICs) face persistent significant challenges in health care services and health research, including resource limitations, workforce shortages, and disease burdens. AI in health care holds significant promise to improve health care quality and efficiency in these settings, particularly by enhancing diagnostics, optimizing resource allocation, and supporting public health interventions. While it would be misleading to suggest that AI can automatically overcome equity gaps, health care AI holds the potential to help address disparities in access and quality—provided it is designed and implemented within equitable and context-sensitive frameworks [1-3].

Debates around decolonizing global AI governance have further drawn attention to the Western-centric nature of existing technological paradigms, calling for inclusive frameworks that integrate local expertise and mitigate structural biases in health care AI [4]. In recent years, digital health innovations such as mobile health have expanded rapidly in LMICs due to the widespread use of mobile devices [1,5], demonstrating both interest and readiness for digital transformation.

Despite its potential, AI in health care and health services research is not impervious to ethical, functional, and social challenges. Different stakeholders, from governments to clinicians, often pursue different goals, with equity being only one among many. The AI lifecycle itself can introduce or amplify inequities through biased data, limited transparency, and accountability gaps. Moreover, privacy and data protection remain central ethical concerns in public health applications [6-9].

For these reasons, AI in health care should prioritize involving and collaborating with humans throughout the entire AI lifecycle in a “human-centered AI” approach to enable transparency and the delivery of equitable outcomes. The approach should also encompass a multidisciplinary approach to expertise [10], involving specialists, ethicists, social scientists, lawyers, frontline health care workers, health scientists, AI or machine learning practitioners, and consumers.

Furthermore, Chow and Li [9] suggest that the refinements of AI models can amplify the biases and create outputs that favor developed countries. Thus, the use of AI must also factor in the cultural, societal, and governance structures, as one’s health depends on their environment, literacy, and health care systems [6]. Yet, most existing literature focuses on barriers to health care AI in high-income countries, leaving a significant gap in understanding the unique challenges and enablers in LMIC settings.

To address these gaps, participatory design (PD) and sociotechnical systems design (STSD) frameworks provide valuable lenses [10-12]. PD is a research approach for developing technological solutions to knowledge gaps, involving users directly in the design and testing of technologies, ensuring that their insights guide system development [12]. Similarly, STSD considers human, social, technical, and organizational factors in the design of technologies [13-15]. The Lancet and Financial Times Commission on Governing Health Futures 2030 [16] highlights the need for inclusive, rights-based digital governance that prioritizes equity, trust, and local engagement—especially for youth growing up in today’s digital world. Moreover, a recent report by Stanford University’s Centre for Digital Health [14] also highlights user-centered design, collaboration, and transparency as key principles for responsible AI deployment in LMICs.

Considering the unique challenges confronting LMICs, our study adopted elements of PD and STSD to elucidate the social, cultural, and political factors influencing health care AI’s development and adoption in these countries. These theoretical concepts help to illuminate the role of governance, power, values, and local knowledge in human-AI interaction, which in LMIC contexts may act not merely as a technical partnership but as a bridge between the global technologies and local contexts.

**Figure 1.**
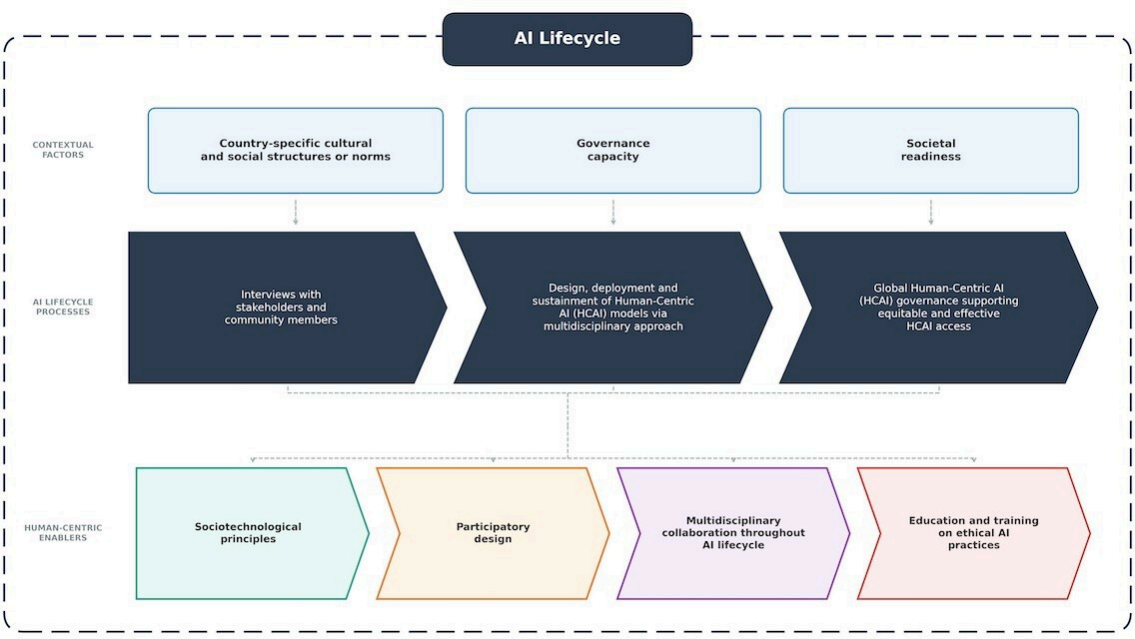
Conceptual framework illustrating intersections of cultural, governance, and societal factors with each stage of the AI lifecycle in LMICs. Human-centric enablers (eg, participatory design and multidisciplinary collaboration) are essential for ethical, inclusive, and locally adaptable AI outcomes. AI: artificial intelligence; LMIC: low- and middle-income country.

Therefore, to curb these challenges in LMICs, our research aimed to (1) understand how human involvement across the AI lifecycle influences the ethical, cultural, and contextual alignment of AI systems in LMICs and (2) examine how cultural, societal, and governance factors shape the perception, development, and deployment of AI in LMIC contexts (Figure 1).

## Methods

### Participant Recruitment

We adopted a purposive sampling method for participant selection, targeting individuals actively engaged in AI health development within LMICs. This led us first to identify 13 countries that displayed significant activity in AI health, assessed by the volume of projects funded by the International Development Research Centre (IDRC) in Canada and the robustness of their research and development sectors. The countries selected are Lebanon, Pakistan, Egypt, Kenya, Uganda, Mexico, Argentina, Bangladesh, Colombia, Tanzania, Indonesia, the Philippines, and Ghana, representing regions across MENA (Middle East and North Africa), Africa, Latin America, and Asia. The inclusion of participants from multiple regions enabled comparative insights across different sociopolitical and health care environments, even though the limited number per country (1‐3 participants) does not allow for country-level generalizations.

Given the funding role of IDRC in the project, our participants’ recruitment was focused on IDRC-funded grantees, chosen for their accessibility and relevance to our research aims. We prioritized participants who had at least 3 years of experience in the domain of AI in health. While this threshold may appear modest, it reflects a pragmatic decision given the relatively recent uptake of AI research and implementation in LMICs. We consider 3 years sufficient for professionals to have encountered ethical, legal, or practical challenges relevant to our study, while ensuring an inclusive and context-appropriate sample.

Participants were contacted via email to schedule interviews. Before each interview, they were requested to sign a consent form that outlined the objectives, procedures, and any potential risks associated with the study. Enrollment was voluntary, and no compensation was offered.

### Data Collection

The interviews were conducted online, via secure Zoom video calls, and both audio and video were recorded. However, only the audio recordings were transcribed for analysis purposes, while the video recordings were deleted shortly after the completion of each interview. In addition, notes were taken during the interviews to provide additional context.

Each interview lasted approximately 60 minutes. To streamline the interview process and facilitate comprehensive discussions, we developed a semistructured interview guide with predefined thematic areas and guiding questions that were adapted to each participant’s expertise and context. The guide was structured around the KAP (knowledge, attitude, and practice) framework [17], consisting of 3 parts: knowledge/experience, attitude, and practice. The guide was piloted and adjusted as needed in response to new information that emerged during the initial interviews.

The majority of interviews were conducted in English and did not require translation. However, in a few cases, particularly with participants from Latin America, interviews were held in Spanish and later translated into English for transcription and analysis. Translation accuracy was verified by bilingual team members to ensure consistency and preservation of meaning.

### Data Analysis

The audio recordings were transcribed verbatim using Adobe Premiere Pro and were deidentified. The quality of the transcriptions was revised manually by the team and compared with the audio recording of each interview. A thematic analysis approach was used for coding and analyzing the transcripts. Given our focus on context-specific perceptions and practices, thematic analysis was an appropriate methodological choice to explore the sociocultural and governance factors shaping AI adoption. Its flexibility allowed us to identify patterns and meaning across diverse experiences, aligning well with our research question. The data were coded using the MAXQDA software. The coding process followed a hybrid approach, combining deductive and inductive strategies: an initial coding framework was developed deductively based on the interview guide and theoretical concepts, while additional codes were created inductively to capture emerging insights from the data. Similar codes were grouped into themes, which were iteratively refined through constant comparison and discussion among the research team to ensure conceptual clarity and coherence across the dataset.

In this study, PD and STSD were used primarily as conceptual and analytical frameworks rather than as full methodological designs. The principles of PD informed the development of the interview guide, which was designed to reflect participants’ perspectives and adapted dynamically to their professional and contextual realities. Similarly, STSD principles informed the interpretation of findings, helping the team consider the interplay between human, organizational, and technical dimensions in the adoption of AI in health. This approach ensured that both the data collection and analysis remained grounded in real-world stakeholder experiences and sociotechnical contexts.

To validate the analysis, two team members separately analyzed the first set of interviews and compared their initial coding results to identify harmonization patterns. The coding tree was continuously adapted to accommodate the insights and needs of all interviews. For the thematic analysis, the team worked collaboratively to analyze the data and construct themes, minimizing individual biases and ensuring a more robust interpretation of the data. Finally, the coding tree was revised to refine and clean the codes, ensuring that they accurately represented the data. This iterative and collaborative process strengthened the reliability and transparency of the final themes.

### Ethical Considerations

Prior to data collection, the study materials were submitted to the Swiss ethics committee for evaluation. In accordance with the Swiss Human Research Act (HRA, article 2, paragraph 1) [18], the committee confirmed that ethical approval was not required, as the research did not involve personal or health-related data. Therefore, no formal ethics board approval number was issued.

All participants provided written informed consent prior to their interviews. The consent form outlined the study’s objectives, procedures, and any potential risks associated with participation. Enrollment was entirely voluntary.

To ensure participant privacy and confidentiality, all audio recordings were transcribed verbatim and deidentified. Video recordings were deleted shortly after the completion of each interview. Only deidentified data extracts are presented in this paper. All data were stored securely and accessible only to the research team. No compensation was provided to participants for their involvement in this study.

## Results

### Overview of Participants and Emerging Themes

Participants were both researchers—working in academia or research institutes and actively involved in AI research—and innovators—working for or owning small startup companies. These two groups provided distinct perspectives on AI ethics perceptions and needs. Table 1 summarizes the characteristics of our participant pool.

**Table 1.** Study demographics (n=21).

Sample	AI experts
Geographical location, n	
Lebanon	2
Egypt	1
Kenya	2
Uganda	1
Tanzania	1
Ghana	2
Mexico	1
Colombia	2
Argentina	2
Indonesia	3
Philippines	1
Bangladesh	2
Pakistan	1
Gender, n (%)	
Women	9 (43)
Men	12 (57)
Sector/role, n (%)	
Researchers	11 (52)
Innovators	10 (48)
Duration, mean (range)	40 minutes (30 minutes to 1 hour)

From the in-depth interviews, 3 themes and 9 subthemes emerged (Table 2), presented through illustrative, deidentified data extracts. The data were diverse and allowed for the detection of differences and capture of needs across various cultures and geographies, allowing for comprehensive results.

**Table 2.** Overview of key themes and their relevance across the AI^a^ lifecycle in LMIC^b^ contexts.

Themes and subthemes	AI lifecycle phases	Key insights
1. Human oversight and ethical considerations in health care AI		
1.1. Integrating the human-in-the-loop concept is essential	Design, development, deployment, evaluation	Human oversight is indispensable for safety, contextual relevance, and trust
1.2. Readiness of local expertise for human oversight	Development, deployment, governance	Limited ethics/regulatory capacity leads to fragmented, reactive oversight
1.3. From ethical practice to continuous compliance	Deployment, evaluation/monitoring, governance	Move beyond checklists to ongoing monitoring and adaptive governance
2. Local collaborative and inclusive AI development		
2.1. Human-centered participatory design through local stakeholder engagement	Problem framing, design, data collection	Co-design with local stakeholders ensures relevance and acceptance
2.2. Multidisciplinary and gender-inclusive teams	Design, development, evaluation	Interdisciplinary, gender-inclusive teams reduce bias and improve fairness
2.3. Fostering partnerships to overcome capacity challenges	Governance, policy translation, ecosystem building	Cross-sector/regional partnerships strengthen capacity and regulatory coherence
3. Designing contextually grounded AI for LMICs		
3.1. Embedding cultural awareness into human-centric AI systems	Design, data curation	Cultural and linguistic adaptation is critical for usability and trust
3.2. Addressing social and structural inequalities through AI	Data, deployment, implementation	Ignoring access and literacy gaps risks amplifying inequalities
3.3. Local understanding of fairness and ethics	Design, governance, evaluation	Fairness and inclusion are locally defined; ethics centers on trust, consent, and usefulness

a AI: artificial intelligence.

b LMIC: low- and middle-income country.

### Theme 1: Human Oversight and Ethical Considerations in Health Care AI

#### Theme 1.1: Integrating the Human-in-the-Loop Concept Is Essential

##### The Indispensable Role of Human Judgment in Health Care AI

A human-in-the-loop (HITL) approach is crucial to prevent AI misinterpretations, ethical violations, and safety risks. AI lacks the nuanced judgment required for complex health care scenarios, such as diagnostic decision-making, mental health interventions, and patient communication. In clinical settings, for example, human professionals must always validate AI-assisted diagnosis to ensure accuracy, ethical compliance, and patient safety [Q1.1.1]. As one participant put it, “...It’s only AI-powered. It’s never 100% AI...” [Q1.1.2], reinforcing the idea that AI should augment rather than replace human expertise.

Human oversight also mitigates bias from nonrepresentative data. In LMICs, models often rely on datasets from high-income settings [Q1.1.3-Q1.1.5]; without local expert review, predictions risk being biased or clinically irrelevant [Q1.1.6, Q1.1.7]. Participants stressed that bench accuracy does not guarantee clinical use: models performing well in controlled tests can falter in real-world deployment due to contextual misalignment and limited adaptability [Q1.1.8-Q1.1.10]. As one noted, “His accuracy scores are 99%...but the utility...is just not that much” [Q1.1.11]. Thus, accuracy must be coupled with contextual validation and ongoing human review.

##### Responsible AI Is the Practical Implementation of Ethics

A recurring theme in the discussions was that responsible AI is more than just ethical principles—it is about “the actions we have to do” throughout the AI development and how to do it [Q1.1.12-Q1.1.16]. To complement the aforementioned, participants described responsible AI as the practical and technical implementation of ethical considerations [Q1.1.17]. Experts highlight the need for concrete accountability measures from the very beginning at all levels of AI development, deployment, and oversight, rather than relying on vague ethical guidelines [Q1.1.18, Q1.1.19].

Transparency and explainability emerged as fundamental requirements for HITL: “The first word that comes to my mind is transparency” [Q1.1.20]. Transparency enables technical validation and trust, and is particularly vital for user-facing health care applications and in regulatory gray zones [Q1.1.21-Q1.1.25]. In such contexts, transparency is a pragmatic path to responsible practice while legal accountability mechanisms evolve.

##### AI Risks Depend on Usage, Not Just Technology

Another key insight from participants was that the risks associated with AI do not stem solely from the technology itself but from how it is used [Q1.1.26-Q1.1.28].

*The technology is not inherently good or bad. It depends on how it’s applied, who controls it, and what values guide its use.*

[Q1.1.29]

This perspective aligns with concerns about AI dependency and misuse, particularly in LMICs where digital literacy is limited. A researcher shared an example of growing dependence: *“*I have students who write an email in English through ChatGPT because they don’t trust what they themselves write,” illustrating behaviors that can translate into overreliance in clinical workflows if unchecked [Q1.1.30]. Participants also called for shared accountability across developers, organizations, and users [Q1.1.31, Q1.1.32].

##### Human Oversight Is the Key to Ethical and Socially Acceptable AI

Finally, participants emphasized that ethical and socially acceptable AI cannot be achieved without meaningful human oversight. They argued that technical performance alone is insufficient; what matters is how AI supports contextual, ethical, and clinically relevant decision-making [Q1.1.33, Q1.1.34]. To ensure ethical and practical outcomes, participants described processes where human experts review AI outputs regularly, adjusting and refining the system based on context and input.

*We do a lot of review of the answers on a day-to-day basis to see what kind of suggestions the AI gives...and whether they need to be reworked, specifically based on the inputs.*

[Q1.1.35]

This illustrates how continuous human evaluation, before and after deployment, is key to ensuring responsible AI use that aligns with societal needs and expectations in health.

### Theme 1.2: Readiness of Local Expertise for Human Oversight

#### Limited Local Capacity and Dependence on Foreign Expertise

Ensuring ethical AI implementation in LMICs requires more than regulatory frameworks; it relies on the presence of local expertise to oversee systems effectively. Yet, many developers, regulators, and researchers in these settings lack formal training in AI ethics and governance, leading to overreliance on foreign experts, weak oversight, and inconsistent ethical assessments, ultimately limiting responsible AI adoption [Q1.2.1, Q1.2.2]. As a researcher stated, “I also feel that, well, the truth is that I lack, well, that knowledge. And without that knowledge, it is very difficult to include it, right?” [Q1.2.3] and as a result, many initiatives remain surface-level [Q1.2.4]. This limited know-how results in reactive rather than proactive monitoring, constraining responsible AI deployment in health care systems:

*We’re mostly reacting to problems as they come up. There’s no structured system to monitor AI before deployment. Institutions are still figuring things out.*

[Q1.2.5]

Due to insufficient in-house expertise, many LMICs rely on external AI experts to develop ethical guidelines and conduct risk assessments [Q1.2.6]. While this facilitates knowledge transfer, it raises concerns about long-term sustainability and local ownership of AI governance [Q1.2.7]. Imported frameworks, often based on Western priorities, may not fit the socioeconomic and health care realities of LMICs [Q1.2.8, Q1.2.9].

Moreover, many AI teams lack confidence in discussing AI ethics. Some participants acknowledged feeling uncomfortable engaging in AI ethics discussions because they see it as beyond their expertise: “I’m probably not comfortable, I’m just guessing” [Q1.2.10]. As a result, AI ethics remains an abstract concept rather than a practical guideline that teams integrate into their development processes. Participants emphasized that addressing this capacity gap requires context-specific training in AI ethics, governance, and regulatory literacy, tailored to local health systems and professional needs.

#### Regulatory Bodies and Research Reviewers Are Underprepared for AI Oversight

One of the most pressing challenges is that regulatory bodies and ethical review committees often lack the technical expertise needed to critically evaluate AI systems [Q1.2.11, Q1.1.12]. A participant from Kenya noted that regulators struggle to keep up with AI innovations: “Innovators are at kilometer ten, and the regulators are just starting to run” [Q1.2.13]. In Mexico, the absence of AI-specific regulations forces companies to operate in legal ambiguity:

*We don’t have a law. So what do you do? You either get approval somewhere else to buy credibility in your market, or you just go ahead and deploy your AI solution because there is no authority to stop you*.

[Q1.2.14]

This gap produces inconsistent approvals; some tools face unnecessary delays while others bypass scrutiny [Q1.2.15, Q1.2.16].

A similar challenge exists within ethical review committees, which are often underprepared to evaluate AI projects from both a technical and ethical standpoint, taking overly cautious approaches. As a researcher noted: “You almost feel like the review boards are trying to be too safe” [Q1.2.17]. Similarly, AI researchers emphasized the difficulty of explaining AI models to ethics review committees [Q1.2.18]. This creates bureaucratic barriers without improving safety [Q1.2.19]. Participants proposed targeted AI training for ethics committee members to strengthen evaluation capacity [Q1.2.20]. Building such expertise locally is essential for meaningful human oversight and safe health care innovation.

### Theme 1.3: From Ethical Practice to Continuous Compliance

#### The Challenge of Ethical AI Compliance in LMICs

AI governance in LMICs often relies on “compliance checklists rather than comprehensive ethical assessments” [Q1.3.1]. While checklists offer a basic framework for oversight, they fail to account for complex AI risks such as bias amplification, data privacy violations, and unintended consequences [Q1.1.17]. Ethical compliance is often treated as a static requirement rather than an evolving process, limiting the effectiveness of oversight mechanisms [Q1.3.2]. This approach creates gaps in continuous monitoring, making it difficult to address emerging ethical concerns.

Instead of systematic governance mechanisms, AI compliance often depends on individual efforts by developers, researchers, or small teams. A participant described the situation:

*Ethical considerations have been, let’s say, a personal and a conscious effort...we don’t have laid-down legislations concerning the ethical use of artificial intelligence. So for me, it’s like I have come up with my principles*.

[Q1.3.3]

Another confirmed that efforts remain fragmented [Q1.3.4]. While some researchers pursue ethical training on their own, the lack of structured support means these efforts often remain informal. This ad hoc approach limits accountability and consistency in health care AI oversight.

#### The Need for Capacity-Building and Regulatory Adaptation

Participants called for targeted capacity-building efforts to reduce reliance on external expertise and develop strong local oversight. While some innovators have taken the initiative to train regulators on AI risks and governance, progress remains slow due to unclear mandates [Q1.3.6]. One AI researcher described this approach:

*...I approach them and say, hey, you are missing this and this and this. And we think we can provide training to you so you can understand better about AI.*

[Q1.3.5]

In several cases, regulators and policymakers lack even a basic understanding of AI, making it difficult to establish meaningful or technically sound policies. As one participant emphasized, “There must be knowledge of all aspects” [Q1.3.7], while others noted that “regulators don’t understand what kind of algorithm you’re going to use” [Q1.3.8], or “don’t understand essential concepts” relevant to AI’s application [Q1.3.9]. Institutionalizing ethical practice—through training, iterative evaluation, and adaptive regulation—is essential to sustain human oversight across the AI lifecycle. This shift from static compliance to continuous learning and monitoring would allow LMIC health systems to build trustworthy, context-aware AI that evolves with local realities.

### Theme 2: Local Collaborative and Inclusive AI Development

#### Theme 2.1: Human-Centered Participatory Design Through Local Stakeholder Engagement

For AI to be ethically sound and socially relevant, it must be co-designed with local communities rather than imposed by external actors. In LMICs, cultural, linguistic, and economic diversity means that top-down AI solutions often fail to address local priorities and realities. Participants emphasized that human-centered PD enables collaboration and trust, ensuring that AI systems reflect local values and needs.

*There are some communities where you have to speak to the chiefs and opinion leaders first...Once they understand the project and agree with it, they can introduce you to the community members so that the project can proceed*.

[Q2.1.1]

Such practices embody PD’s principle of *cocreation*, fostering ownership and legitimacy in health care innovation.

Participants argued that participation must go beyond consultation toward shared decision-making and accountability. Otherwise, AI risks reinforcing structural inequalities when frameworks are designed externally and fail to include those most affected by deployment [Q2.1.2-Q2.1.4]. Meaningful participation, they argued, requires collaboration between government, academia, and civil society to ensure that AI technologies are *context-responsive and ethically grounded* [Q2.1.5, Q2.1.6].

However, meaningful participation also depends on education, capacity-building, and digital literacy. Without investment in local training and infrastructure, participatory efforts risk becoming tokenistic rather than truly inclusive. As one participant noted:

*You can develop the best systems, but if the education and training don’t go well, people won’t use the technologies because they do not understand them or appreciate why they should use them*.

[Q2.1.7]

In this sense, participatory design and capacity-building are interdependent; local involvement drives understanding, and understanding sustains long-term adoption.

#### Theme 2.2: Multidisciplinary and Gender-Inclusive Teams

##### The Value of Multidisciplinary Collaboration

Developing fair and contextually relevant AI systems requires a multidisciplinary collaboration that integrates technical, ethical, and social expertise. AI is not just a technological tool but a sociotechnical system shaped by human values, societal norms, and governance structures. Ensuring ethical implementation means involving computer scientists, ethicists, health care professionals, and social scientists to anticipate risks, mitigate biases, and promote inclusivity [Q2.2.1, Q2.2.2]. In LMICs, where AI adoption is still evolving, such collaboration is essential to align AI with local realities. A participant emphasized this need:

*Our team itself is made up of three different people. I am from a computer science background, my two other colleagues, one is from sociology, and the other is from social work. Because of our various backgrounds, we bring that expertise to the board to make sure that every system is evaluated*.

[Q2.2.3]

However, participants acknowledged that many AI teams in LMICs lack expertise in ethics, responsible AI, and regulatory compliance, which limits their ability to foresee ethical or legal challenges. As one participant explained, “One of the biggest challenges is the lack of awareness about ethical considerations, data management, and responsible AI principles” [Q2.2.4]. Without cross-disciplinary collaboration, AI systems risk bias, misinterpretation, and regulatory failure [Q2.2.5].

##### Gender Balance as an AI Governance Consideration

Gender diversity emerged as another critical yet underaddressed aspect of inclusive AI governance. Teams remain predominantly male, limiting the diversity of perspectives in both design and leadership.

Most of the people implementing AI projects are women, but the databases and training datasets are overwhelmingly created by men, which skews the outputs.

[Q2.2.6]

Others described structural barriers restricting women’s participation in research and policy [Q2.2.7] and observed that gender quotas sometimes serve symbolic rather than substantive purposes [Q2.2.8].

Participants highlighted that when women and other marginalized groups are excluded, their health needs and lived experiences are also omitted from AI design and evaluation. This results in inequitable systems and poorer health outcomes [Q2.2.9]. Several participants extended this concern beyond gender, underscoring the importance of socioeconomic and disciplinary diversity [Q2.2.10]. Ensuring gender and social inclusivity within AI teams is thus not only a question of representation but a precondition for ethical, relevant, and effective innovation.

### Theme 2.3: Fostering Partnerships to Overcome Capacity Challenges

Participants highlighted that effective AI governance in LMICs requires sustained collaboration across national, regional, and international levels. While regional initiatives exist to harmonize AI policies, they are often hindered by institutional fragmentation and unclear mandates. One participant described their effort to engage with regulatory bodies:

*We wanted to provide training to regulators on what they can regulate, what the risks are, and what they need to consider. Unfortunately, many of the regulators themselves didn’t know who was responsible for AI regulation. It took us six or seven months just to get one agency to start talking...*

[Q2.3.1]

Participants also described how gaps in technical knowledge complicate regulatory review processes. In some cases, agencies apply frameworks intended for hardware to AI software systems, creating confusion and delays [Q2.3.2]. Another participant noted: “Innovators are at kilometer ten, and the regulators are just starting to run” [Q2.3.3].

Participants also highlighted that in many LMICs, AI regulation remains a low policy priority, contributing to inconsistent oversight and delayed implementation [Q2.3.4-Q2.3.6]. Strengthening institutional capacity thus requires collaborative partnerships between policymakers, AI developers, health care professionals, and regional networks. These alliances can promote knowledge exchange, harmonized regulatory standards, and joint training efforts, enabling regulators to keep pace with rapid technological advances.

### Theme 3: Designing Contextually Grounded AI for LMICs

#### Theme 3.1: Embedding Cultural Awareness Into Human-Centric AI Systems

##### Cultural Alignment as a Prerequisite for AI Acceptance

AI is not a neutral technology; it reflects the values, priorities, and knowledge of its developers. In LMICs, where cultural, social, and governance factors shape both perception and use, aligning AI with local realities is essential for ethical and effective implementation. Our findings reveal that AI development and adoption are deeply shaped by human factors, including cultural expectations, social hierarchies, and local understandings of ethics. Participants consistently emphasized that AI systems must be developed with cultural awareness and inclusivity, as these determine usability, trust, and social acceptance. As one specifically asked: “Does the solution you’re going to develop respect the culture and tradition of that particular region?” [Q3.1.1]. Without such alignment, AI risks exclusion, misinterpretation, and rejection by the very communities it aims to serve.

##### Language Barriers to AI Inclusion

A significant barrier to equitable AI is the language gap. Many AI models are designed primarily for English-speaking populations, leading to poor performance in local languages and cultural misalignment. A researcher emphasized:

*So people need to understand, what’s the capacity of an AI model? And, what it can do, what it cannot do, and you cannot just transfer cultural knowledge in those scenarios...*

[Q3.1.2]

This challenge extends beyond linguistics to social norms and values, affecting AI usability and trust in technology [Q3.1.3]. A researcher noted:

*A lot of the language models that work well in English-speaking populations fail to interpret ideas in our language and for our people. This is where the transfer of cultural knowledge becomes essential*.

[Q3.1.4]

##### Cultural Taboos and Gender Roles

Beyond language, participants described how cultural and social expectations, especially around the topic of reproductive and mental health, can limit the effectiveness of AI systems. One participant explained when discussing maternal health in Muslim societies:

*People are, let’s say, not open about it. Okay? Right. People don't come forward and talk about these issues*.

[Q3.1.6]

Similarly, cultural misalignment in AI governance frameworks was raised by another participant:

*Culture is one thing that we uphold, and we don’t want technologies that tend to represent our culture in a negative light. Cultural sensitivity is paramount*.

[Q3.1.5]

AI tools designed without such sensitivity often fail when deployed in new contexts [Q3.1.7], underlining the need for continuous cultural adaptation throughout the AI lifecycle.

Several participants also highlighted the impact of patriarchal structures, where male dominance shapes access to education, health care, and decision-making. One participant described the difficulty of engaging women in health care AI solutions: “...people don’t really have anything to go towards, so women don’t prioritize their health” [Q3.1.8]. Women’s participation in AI research and development is also constrained by societal expectations [Q3.1.9], and design choices frequently reflect male-dominated perspectives [Q3.1.10]. In some cases, gender-specific AI solutions can unintentionally reinforce exclusion. A participant working on a women-focused AI project noted: “But we are a product for women, so there’s not really room for men” [Q3.1.11].

To counteract these issues, participants stressed the need for gender-inclusive datasets and participatory approaches.

*And just because something doesn’t look significant to someone, you cannot just exclude them from your discussion and your research*.

[Q3.1.12]

Embedding diversity and human oversight in AI development was viewed as essential to ensuring that technologies reflect, rather than distort, the social realities of their users.

### Theme 3.2: Addressing Social and Structural Inequalities Through AI

#### Western-Centric Assumptions and Local Realities

Beyond culture, participants discussed how AI often reflects, and can entrench, underlying inequalities in LMIC contexts. These include digital divides, gendered access to care, and structural biases in datasets and deployment strategies. AI solutions are frequently built on Western-centric assumptions that fail to account for the realities of LMICs. Local contexts, shaped by legal frameworks, education levels, digital literacy, and social hierarchies, profoundly influence how AI is perceived and used. As one participant noted:

*Something that may be legal in one country may be illegal in mine. The frameworks around AI governance cannot be one-size-fits-all*.

[Q3.2.1]

#### Gender and Social Bias in AI Development and Deployment

AI systems frequently inherit the biases of their creators, reproducing existing inequalities related to gender, class, race, and access to resources. One researcher observed how gender norms affect AI adoption:

*Women will be able to talk to other women about these issues because, in our country, women are not comfortable talking with men about their problems*.

[Q3.2.2]

Similarly, others emphasized that gender-based violence and reproductive health are deeply tied to cultural taboos, complicating AI’s capacity to address them [Q3.2.3].

These embedded biases also extend to racial and socioeconomic disparities. A participant asked:

*Do AI systems differentiate individuals based on their skills, or do they differentiate based on their skin color and ethnicity?*

[Q3.2.4]

Even models designed to be objective often fail to correct systemic inequalities, tending instead to benefit urban, affluent users while marginalizing those with limited digital access [Q3.1.4].

#### Digital Literacy and Unequal Access

Participants stressed that AI technologies are often developed with digitally literate, urban, and affluent users in mind, overlooking the substantial portions of LMIC populations who lack access to the internet, electricity, or necessary devices. As one participant reflected, “Innovations tend to, especially when it comes to AI, inadvertently [be] made for people who already have access” [Q3.2.5]. They added that “60%‐70% of the population in [their country] does not have regular internet access” [Q3.2.6], making AI inaccessible to the majority.

Infrastructure limitations further compound this divide:

*There will be a bias...public hospitals are not going to have access to the technology that is essential to be able to later analyze the image.*

[Q3.2.7, Q3.2.8]

Even basic digitalization steps, like scanning or data storage, remain unaffordable in many facilities. Others noted that AI systems often assume a baseline of digital literacy, excluding users without education or exposure to digital tools:

*We try to close the gap, but if we base systems on AI...what’s going to happen in places that don’t have access to internet or don’t have electricity? Is this giving some people more of an advantage and other people are just being left behind?*

[Q3.2.9]

This digital divide is compounded by linguistic barriers, as many systems are designed for English-speaking populations, making them even less usable in local contexts with low digital and language literacy.

#### AI’s Potential to Reduce Inequalities

Despite its potential for bias, AI also holds the promise of mitigating human errors and improving equity when thoughtfully designed and guided by human oversight. One participant described how AI can help reduce inconsistency in medical decision-making:

*You find that there is a huge difference between the opinion of radiologists in the same hospital. So, the good thing about AI, is it captures this automatically, and it resolves these discrepancies*.

[Q3.2.10]

Another emphasized that AI should be used intentionally to reduce inequality rather than reinforce it [Q3.2.11]. Achieving this goal requires localized data, participatory design, and improved digital access. Participants agreed that human oversight and inclusivity must remain central to ensure AI supports, rather than amplifies, existing disparities.

### Theme 3.3: Local Understanding of Fairness and Ethics

#### Contextual and Relational Ethics in LMIC Settings

A recurring insight from participants was that global AI ethics principles, while helpful, often fail to capture locally meaningful understanding of fairness, trust, and social responsibility. In LMICs, ethics is frequently grounded in relational and contextual judgments rather than universalist definitions.

#### Blurred Lines Between Fairness and Inclusion

Global frameworks tend to define fairness and inclusion as distinct concepts, yet participants revealed that these principles are often intertwined in practice, especially in LMICs. As one participant from Uganda explained:

*I find it’s a little bit of a blah in my mind between responsible AI, gender ethics and so gender equity and inclusion and ethics*.

[Q3.3.1]

Another asked:

*Is it clear the difference between fairness and inclusion? No, I can combine them*.

[Q3.3.2]

This convergence reflects how fairness in LMIC contexts is understood not as a mathematical balance in datasets but as ensuring AI serves communities equitably, considering historical, social, and economic disparities [Q3.3.3, Q3.3.4]. These perspectives suggest that fairness and inclusion are experienced together, as part of a broader commitment to social justice and contextual relevance.

#### Local Ethical Emphasis on Consent, Trust, and Usefulness

When asked about AI ethics, many participants instinctively focused on data-related issues, such as privacy, consent, and ownership. One noted:

*AI is providing data for the person who will be affected by this data*.

[Q3.3.5]

Others linked fairness to data bias, emphasizing that unrepresentative datasets perpetuate inequality across domains such as health and gender [Q3.3.6-Q3.3.8]. Ethical debates in many settings remain focused on compliance and data protection [Q3.3.9-Q3.3.11], often overlooking broader questions of purpose, trust, and social benefit.

In addition to data considerations, participants highlighted trust and accountability as key values in their understanding of AI ethics. A researcher emphasized the importance of prioritizing human needs in AI development:

*...is it helping the user? Is it actually benefiting the user? whether they know it’s AI or not...whether they understand what the technology implications are...is the user’s life better? with the use of AI as opposed to that baseline?*

[Q3.3.12]

Another participant from Indonesia noted that responsible AI development should not only be about regulatory compliance but also about ensuring AI systems align with moral and societal expectations [Q3.3.13].

Lastly, participants agreed that human oversight remains irreplaceable in ensuring AI aligns with ethical and social values, and local expectations. A participant said:

*In a sense, intellectually, the only thing we can have is the EQ, because we do have emotions. And if you keep championing IQ then you’re going to lose it anyway. GPT is so much smarter than I am.*

[Q3.3.14]

A participant underscored the necessity of keeping human experts involved in AI-assisted decision-making:

*...I would always put a human behind it. That is my solution. It’s only AI-powered. It’s never 100% AI...because we don’t know the nuance of each question*.

[Q3.3.15]

These reflections reinforce that AI should act as a decision-support tool rather than an autonomous decision-maker, ensuring that human judgment, ethical considerations, and contextual understanding remain central to AI’s deployment in LMICs.

## Discussion

### Principal Findings

This study examines the critical role of human involvement in AI development and deployment, focusing on how cultural, societal, and governance factors shape perceptions and expectations of AI in LMICs. Through 21 interviews conducted across 4 regions, MENA, Africa, Latin America, and Asia, five key themes emerged: (1) the necessity of human oversight and the readiness required to support it, (2) the pressing need for AI ethics training, (3) the importance of developing AI systems tailored to local realities, (4) the role of human-centered AI governance, and (5) the value of securing multidisciplinary teams. These findings align with and extend global frameworks, such as the World Health Organization’s (WHO) Ethics and Governance of AI for Health (2021) [19] and UNESCO’s (United Nations Educational, Scientific and Cultural Organization) Recommendation on the Ethics of AI (2021) [20], which call for inclusive, transparent, and contextually grounded approaches to AI governance.

Our results also extend work in science and technology studies that conceptualize AI as a sociotechnical system embedded in local norms and power structures. The findings resonate with the concept of “sociotechnical imaginaries”—collectively held visions of what AI should do, who should control it, and whom it should serve—and echo decolonial AI scholarship that calls for local participatory governance and epistemic diversity [10,21].

Table 2 summarizes the 3 themes and 9 subthemes from the thematic analysis and maps them to the AI lifecycle phases (problem framing/design, data curation, development, deployment, evaluation/monitoring, and governance).

### Bridging Ethical Readiness and Human Oversight

AI development in LMICs is advancing rapidly, yet many researchers and innovators lack the necessary training in AI ethics, bias mitigation, and responsible governance. This knowledge gap limits their ability to anticipate ethical risks, integrate fairness and transparency into AI models, and ensure their solutions align with local societal needs [10]. Similar challenges are documented by the International Telecommunication Union (ITU)/WHO Focus Group on AI for Health, which highlights the absence of technical and regulatory expertise as a barrier to ethical implementation [22].

While AI governance frameworks often focus on national or global policy, the practical challenge lies in equipping AI teams with the skills to embed ethics in daily practice. Without structured training, a total product life cycle approach, and local oversight, ethical considerations remain secondary to technical development. As participants noted, reliance on external datasets and imported governance models often leads to biased outcomes that fail to serve local populations [10,23].

Bridging this readiness gap requires interdisciplinary education programs that combine technical, ethical, and regulatory perspectives. Universities and research institutes should integrate AI ethics modules into curricula and foster partnerships between developers, clinicians, and policymakers. As UNESCO emphasizes, sustainable AI governance depends on “ethical capacity-building and inclusivity at every stage of the AI lifecycle” [20].

### Building Collaborative and Participatory AI Ecosystems

Our findings confirm that collaborative and participatory approaches are central to contextually relevant AI design. For AI to be effective in LMICs, it must be developed with a deep understanding of local realities rather than replicating Western-centric models [10]. A systematic review and thematic analysis [10] discuss the use of health care AI in LMICs and in bridging the global health gap. The “AI deployment paradox” is the obvious risk that AI will aggravate social inequalities in LMIC settings due to the inequalities in digital health, health systems, and social structures. Engaging local stakeholders—policymakers, health care professionals, and communities—helps ensure that AI serves societal needs rather than external priorities [24,25]. This aligns with WHO’s 2021 call for “AI systems that are human-centred by design and co-created with end users.”

However, many participatory efforts in LMICs remain limited by a lack of training, infrastructure, or inclusive representation. Women and marginalized groups, for instance, remain underrepresented in AI governance and design teams [26,27]. As participants described, this gender imbalance can skew datasets and decision-making processes, reflecting structural inequities rather than addressing them [26,27].

To move from tokenism to meaningful inclusion, LMICs must strengthen cross-sectoral collaboration—between academia, government, private sector, and civil society—and adopt participatory design methods that empower users as cocreators. This echoes the PD and STSD frameworks used in our study, where multistakeholder collaboration was seen as essential to align AI with social realities and build public trust [28-30].

However, as several participants emphasized, collaboration alone is insufficient if power and decision-making remain concentrated in external actors or funding bodies. Many described how international projects often impose predefined goals, datasets, or ethical frameworks that fail to reflect local priorities—a dynamic that echoes broader patterns of algorithmic coloniality and dependency [31,32]. True decolonial AI governance, as envisioned by participants, requires redistributing authority and resources toward local researchers, policymakers, and communities, enabling them to define what “ethical” and “responsible” AI means within their own contexts. This involves locally led agenda setting, community-driven data governance, and equitable North-South partnerships that move beyond compliance toward epistemic and structural autonomy in AI development [31,32].

### Embedding Context and Ethics Into AI Lifecycle

AI is not just a technological tool. It is a sociotechnical system shaped by human values, biases, and societal structures [30,31,33]. It also reflects the values, priorities, and assumptions of its creators. In LMICs, ethical implementation requires that AI be embedded in local cultural and social contexts, respecting linguistic diversity, belief systems, and governance structures [10,27,32]. In Africa, for instance, the existence of multiple local languages makes AI implementation difficult, as many systems rely on dominant global languages. A lack of linguistic inclusivity risks excluding significant portions of the population from AI-driven services [23,24]. Similarly, in Asia, cultural taboos surrounding gender-based violence and reproductive health affect how AI can be used to provide information on these sensitive topics [26,27]. AI systems that fail to account for these social constraints risk either being underutilized or reinforcing existing biases. Beyond cultural adaptation, structural barriers such as digital illiteracy and limited awareness of AI further complicate responsible deployment [28]. Many individuals in LMICs struggle with understanding data privacy, algorithmic decision-making, and AI-driven processes, making informed consent particularly challenging. This is especially concerning in health care applications, where individuals may not fully grasp how their data is used or how AI-derived recommendations impact their treatment.

Beyond cultural fit, participants emphasized that fairness and inclusion are interpreted relationally—rooted in trust, consent, and social responsibility rather than abstract principles. This aligns with broader decolonial and feminist technology scholarship, which critiques the universalization of Western ethical standards [10,32]. As one participant observed, fairness in practice means ensuring AI “serves people equitably and does not leave others behind.”

Our findings therefore support global recommendations calling for context-sensitive AI governance that connects ethical principles with real-world implementation [10]. WHO and UNESCO have both underscored the need to translate global norms into local governance mechanisms through participatory frameworks, capacity-building, and accountability systems [19,20].

Furthermore, AI governance must be participatory, meaning communities should co-design AI solutions rather than merely adapting to pre-existing models. Ethical AI is not just about technical safeguards; it requires human judgment, lived experiences, and social awareness to ensure it serves all populations equitably. By embedding human oversight into AI development and deployment, LMICs can leverage AI as a force for innovation while ensuring that it remains aligned with societal needs and ethical considerations.

### Strengths and Limitations

To the best of our knowledge, our research is currently the only qualitative study exploring the human-AI interaction from an ethical perspective in LMICs, given that most published works adopt review methodologies as scoping reviews and systematic reviews, for example [5,34-36]. While our study supported the current positions regarding health care AI in LMICs, such as interdisciplinary approaches unique to each country or region, we also provided deeper, novel perspectives on the existing biases as informed by the local scientists themselves.

While research papers have supported the importance of HITL and the gaps in human-AI collaboration [37,38], there is a lack of empirical evidence, especially in the context of LMICs. Given the thematic analysis methodology, we collected rich data and conducted an in-depth exploration of the views and insights of a wide variety of experts, including researchers and innovators. The multiregional data collection allowed us to reach a first conclusion regarding the different needs, but further research is needed to fundamentally support our insights. Toward the same direction, while our study demonstrates the ethical and practical challenges during AI’s development and deployment, as qualitative research, our results cannot be generalized outside the study population, and more research is needed to explore whether similar insights can be obtained in other areas.

While our sample included a considerable number of research participants from different regions, we may not have captured the conceptions of health care AI researchers in LMICs, which may not be as prominent in the AI field. Given that AI in health care is a growing field in LMICs, a more diverse and inclusive sample would have made the findings more generalizable across most, if not all, LMICs.

Moreover, as this study is part of a research project funded by the IDRC in Canada, participant recruitment was shaped by the project’s focus on sexual, maternal, and reproductive health. This may have introduced biases in how certain aspects of AI ethics and development were discussed. Additionally, the majority of interviewed innovators and startup CEOs were women, likely reflecting a heightened sensitivity to the topic and a gender bias or skew [39]. Given the nature of the subject, cultural considerations inherently played a significant role, potentially influencing participants’ perspectives on AI ethics and human factors in AI development [40].

Lastly, the study team acknowledges the inherent challenges of conducting cross-context research from an external vantage point and actively reflects on these positionality limitations during our analysis and reporting. Although none of the authors are affiliated with the LMIC-based institutions interviewed in this publication, our work is deeply centered around LMIC contexts, informed by ongoing collaborations and research with AI researchers, innovators, and other relevant stakeholders based in diverse LMIC settings. This project was conceptualized by HealthAI—The Global Agency for Responsible AI in Health, an organization with a mission to promote equitable access to AI health innovations globally through the collaborative implementation of AI governance, regulatory mechanisms, and global standards, with a strong focus on LMICs. In keeping with this mission, our study integrates the lived experiences, ethical considerations, and perspectives shared by local stakeholders in LMICs, ensuring that their voices are central to our analysis and insights. We reflect on the limitations of our positionality as external researchers and the importance of ongoing partnerships with LMIC stakeholders.

### Conclusions

This study contributes to the growing literature on responsible and ethical AI by revealing how human oversight, local expertise, and participatory design shape AI implementation in LMIC health care systems. The results demonstrate that human involvement is indispensable across the AI lifecycle for ensuring accuracy, fairness, and contextual relevance.

Policymakers should prioritize AI ethics and regulatory capacity training, participatory governance, and contextually adaptive frameworks that integrate cultural and institutional realities. These priorities echo global guidance from WHO [19] and UNESCO [20], which stress the need for locally led innovation and equitable digital transformation.

For practitioners, embedding human oversight and ethical reflection into AI design ensures that technology complements rather than replaces human judgment. For researchers, our findings highlight the importance of interdisciplinary collaboration that brings together computer science, ethics, law, and social sciences.

Ultimately, grounding AI in local realities is essential not only for equitable health care but also for building global ethical resilience—ensuring that the future of AI is shaped by the diversity of human experience rather than the dominance of a single worldview.

## Abbreviations

AI: artificial intelligence
HITL: human in the loop
HRA: Swiss Human Research Act
IDRC: International Development Research Centre
ITU: International Telecommunication Union
KAP: knowledge, attitude, and practice
LMIC: low- and middle-income country
MENA: Middle East and North Africa
PD: participatory design
STSD: sociotechnical systems design
UNESCO: United Nations Educational, Scientific and Cultural Organization
WHO: World Health Organization
